# List of Graduates of the College of Physicians and Surgeons, Keokuk, Session of 1867–8

**Published:** 1869-02

**Authors:** 


### LIST OF GRADUATES OF THE COLLEGE OF PHYSICIANS AND SURGEONS, KEOKUK, SESSION OF 1867-8.

| *Names of Graduates.* | *Residence.* | *Subject of Thesis.* |
|---|---|---|
| Armstrong, John B | Cincinnati, Iowa | Duties of the Accoucheur. |
| Allin, C. W | Jacksonville, Mo | Scarlatina. |
| Babcock, D. C | Milton, Wis | Pleuritis. |
| Bergen, S. N | Lancaster, Mo | Enteric Fever. |
| Brown, J | Plum River, Ills | Neuralgia. |
| Campbell, A. J | Longwood, Ontario | Hernia. |
| Crawford, James | Dowdsville, Iowa | Intermittent Fever. |
| Dayton, A. O | Hickory Ridge, Ills | Inflammation. |
| Gillett, W. J | Cuba, Ills | Technical Phrases. |
| Glynn, Thos. F | Davenport, Iowa | Duties of the Physician. |
| Gifford, D | Lynnville, Ills | Gastritis. |
| Hayden, H. R | Libertyville, Iowa | Pneumonitis. |
| Knowles, H. H | Castalia, Iowa | Midwifery. |
| Myler, J. M | French Island, Ind | Small Pox. |
| Martin, W. H | Colony, Mo | Pulse, in Disease and Health. |
| McKee. L. D | St. Francisville, Mo | Billious Remittent Fever. |
| McCutchan, J. F | North Henderson, Ill. | Inflammation. |
| Ott, William | Washington, Iowa | Indigestion. |
| Parish, W. O | Pella, Iowa | Rheumatism. |
| Sala, O. P | West Point, Iowa | The Circulation. |
| Sturgeon, W. P | Edgington, Ills | Cerebro Spinal Meningitis. |
| Sanders, S. F | Bushnell, Ills | Physiology of Sight. |
| Tate, J. S | Mexico, Mo | Rheumatism. |
| Williams, John S | Otterville, Ills | Acute General Peritonitis. |

Honorary Degrees.—A. E. McNeall, Bowensburg, Ills.; A. B. McNeall, Columbus, Ills.; W. H. Calkins, Wyoming, Iowa; John Charlton, Freeport, Ills.; Joseph McReynolds, Colony, Mo.; Clinton Fisher, Jacksonville, Ills.

Degree ad Eundem.—A. S. Tinsman, St. Francisville, Mo.

Whole number of Matriculants, 120. Number of graduates, 31.

